# Supplementary material for: Stretchable Nanofiber-Based Felt as a String Electrode for Potential Use in Wearable Glucose Biosensors
Source: Sensors (Basel). 2024 Feb 17;24(4):1283. doi: 10.3390/s24041283 (PMC10891505; doi:10.3390/s24041283)
Supplement: Supplementary file 1 [file sensors-24-01283-s001.zip › sensors-2845085-supplementary.pdf]

Supplementary Material

Stretchable Nanofiber Based Felt as a String Electrode for Potential Use in Wearable Glucose Biosensors

Bianca Seufert, Sylvia Thomas and Arash Takshi *

Department of Electrical Engineering, University of South Florida, 4202 E. Fowler Avenue, Tampa, FL 33620, USA; bseufert@usf.edu (B.S.); sylvia@usf.edu (S.T.)
* Correspondence: atakshi@usf.edu; Tel.: +1-813-974-1121

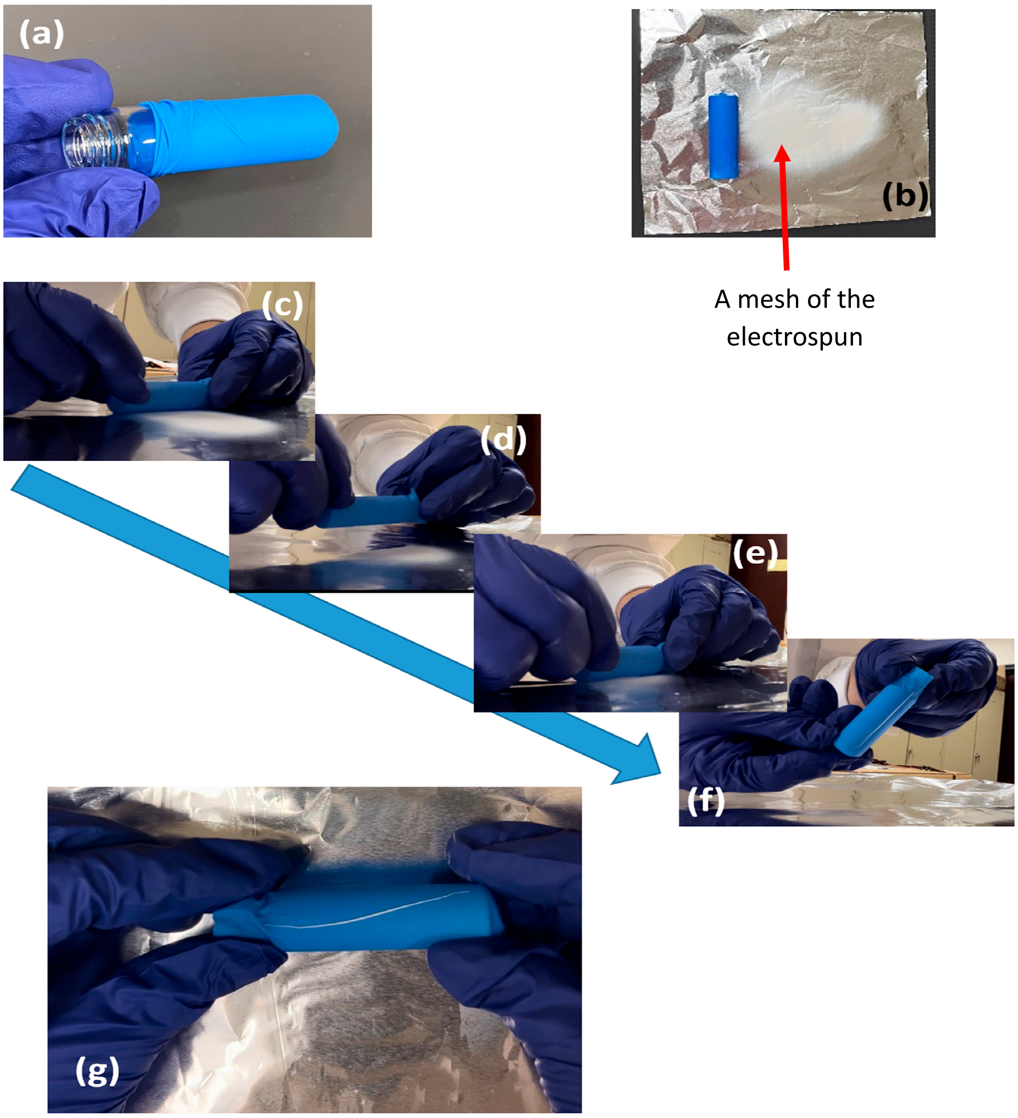

Figure S1: The fabrication process of making NF-Felt from the electrospun PVDF-HFP nanofibers. (a) The roller was made by using a piece of latex rubber on a glass vial. (b)shows a mesh of PVDF-HFP nanofibers on the aluminum collector. (c-f) The roller was manually pressed over the mat and as it was moved forward, it was rotated upward. (g) the NF-Felt string on the roller.

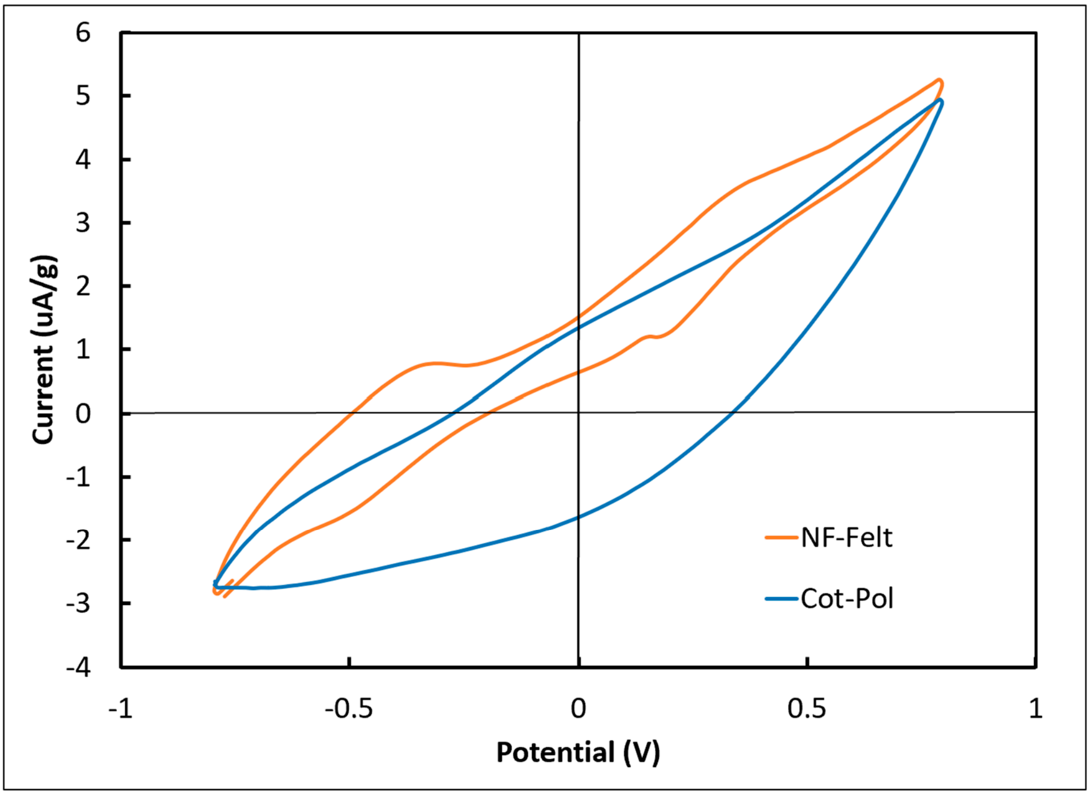

Figure S2: Cyclic voltammetry response comparison between Cot-Pol string electrode and NF-Felt string electrode.

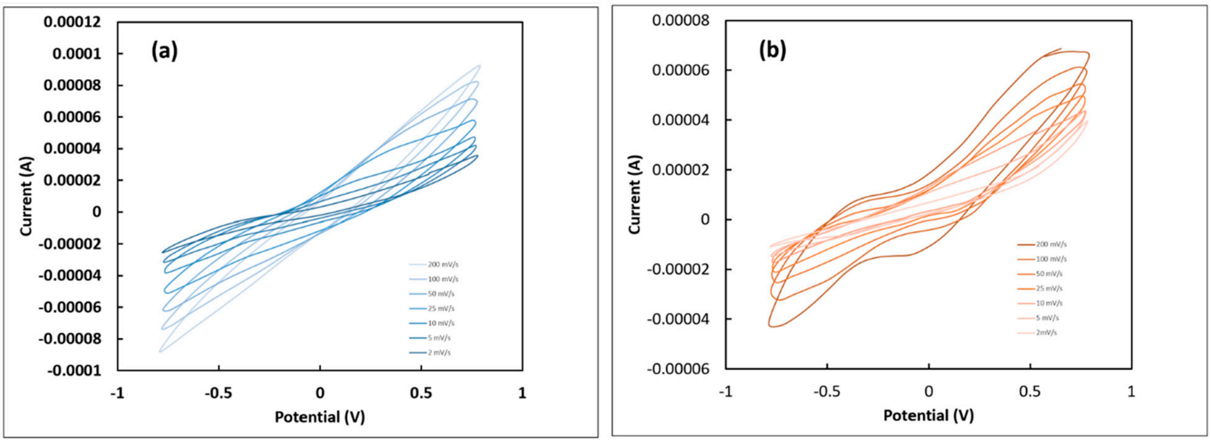

Figure S3: (a) Cyclic voltammetry response of Cot-Pol string electrode at varying scan rates (b) Cyclic voltammetry response of NF-Felt string electrode at varying scan rates.
